# Inhibition effect of copper-bearing metals on arterial neointimal hyperplasia via the AKT/Nrf2/ARE pathway *in vitro* and *in vivo*

**DOI:** 10.1093/rb/rbae042

**Published:** 2024-04-16

**Authors:** Peng Wang, Xiaohe Xu, Guisong Gu, Qianwen Guo, Yanzhi Rao, Ke Yang, Tong Xi, Yonghui Yuan, Shanshan Chen, Xun Qi

**Affiliations:** Department of Interventional Therapy, The First Hospital of China Medical University, Shenyang 110001, China; Department of Ophthalmology, Shengjing Hospital of China Medical University, Shenyang 110004, China; Shi-Changxu Innovation Center for Advanced Materials, Institute of Metal Research, Chinese Academy of Sciences, Shenyang 110016, China; Department of Interventional Therapy, The First Hospital of China Medical University, Shenyang 110001, China; Key Laboratory of Diagnostic Imaging and Interventional Radiology of Liaoning Province, Department of Radiology, The First Hospital of China Medical University, Shenyang 110001, China; Department of Interventional Therapy, The First Hospital of China Medical University, Shenyang 110001, China; Key Laboratory of Diagnostic Imaging and Interventional Radiology of Liaoning Province, Department of Radiology, The First Hospital of China Medical University, Shenyang 110001, China; Shi-Changxu Innovation Center for Advanced Materials, Institute of Metal Research, Chinese Academy of Sciences, Shenyang 110016, China; Shi-Changxu Innovation Center for Advanced Materials, Institute of Metal Research, Chinese Academy of Sciences, Shenyang 110016, China; Liaoning Cancer Hospital & Institute, Clinical Research Center for Malignant Tumor of Liaoning Province, Cancer Hospital of China Medical University, Shenyang 110042, China; Shi-Changxu Innovation Center for Advanced Materials, Institute of Metal Research, Chinese Academy of Sciences, Shenyang 110016, China; Department of Interventional Therapy, The First Hospital of China Medical University, Shenyang 110001, China; Key Laboratory of Diagnostic Imaging and Interventional Radiology of Liaoning Province, Department of Radiology, The First Hospital of China Medical University, Shenyang 110001, China

**Keywords:** copper-bearing metal, neointimal hyperplasia, vascular smooth muscle cells, AKT/Nrf2/ARE pathway, stent

## Abstract

In-stent restenosis can be caused by the activation, proliferation and migration of vascular smooth muscle cells (VSMCs), which affects long-term efficacy of interventional therapy. Copper (Cu) has been proved to accelerate the endothelialization and reduce thrombosis formation, but little is known about its inhibition effect on the excessive proliferation of VSMCs. In this study, 316L-Cu stainless steel and L605-Cu cobalt-based alloy with varying Cu content were fabricated and their effects on surface property, blood compatibility and VSMCs were studied *in vitro* and *in vivo*. CCK-8 assay and EdU assay indicated that the Cu-bearing metals had obvious inhibitory effect on proliferation of VSMCs. Blood clotting and hemolysis tests showed that the Cu-bearing metals had good blood compatibility. The inhibition effect of the Cu-bearing metals on migration of cells was detected by Transwell assay. Further studies showed that Cu-bearing metals significantly decreased the mRNA expressions of *bFGF*, *PDGF-B*, *HGF*, *Nrf2*, *GCLC*,* GCLM*,* NQO1* and* HO1*. The phosphorylation of AKT and Nrf2 protein expressions in VSMCs were significantly decreased by Cu-bearing metals. Furthermore, it was also found that SC79 and TBHQ treatments could recover the protein expressions of phospho-AKT and Nrf2, and their downstream proteins as well. Moreover, 316L-Cu stent proved its inhibitory action on the proliferation of VSMCs *in vivo*. In sum, the results demonstrated that the Cu-bearing metals possessed apparent inhibitory effect on proliferation and migration of VSMCs via regulating the AKT/Nrf2/ARE pathway, showing the Cu-bearing metals as promising stent materials for long-term efficacy of implantation.

## Graphical Abstract

**Figure:**
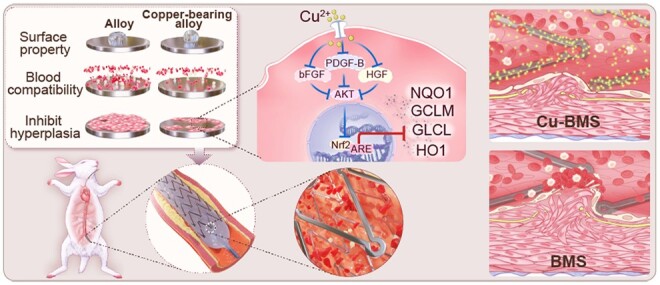

## Introduction

Arteriosclerosis obliterans (ASO) of the lower extremity refers to the progressive stenosis or occlusion of the lumen in lower extremity arteries, which is often manifested as cold and numbness of the limbs, intermittent claudication and resting pain. In severe cases, ulcers and gangrene may occur, which may eventually lead to amputations or deaths [1]. The incidence rate of atherosclerosis is the third in all cardiovascular diseases, and second only to coronary artery disease and stroke. From 2000 to 2010, the number of patients with lower extremity ASO increased by 23.5%. Up to 2010, there were ∼200 million patients with peripheral artery diseases worldwide [2]. Interventional therapy based on stent implantation has become the first choice for ASO treatment, especially for long-segment occlusive lesions [3]. Nevertheless, the emergence of in-stent restenosis (ISR) seriously affects the medium- and long-term efficacies of interventional therapy [4].

Increasing numbers of studies have shown that the mechanism of restenosis includes the following aspects [5, 6]: (i) vascular endothelial cells are damaged during stent implantation; (ii) activation, proliferation and migration of vascular smooth muscle cells (VSMCs); and (iii) platelets aggregate to form thrombosis. Among them, the vascular intimal hyperplasia caused by excessive proliferation of VSMCs is one of the main reasons for the failure of interventional therapy [7]. Drug-eluting stents (DESs), such as polymer-based sirolimus-(Cypher) and paclitaxel-(Taxus) drug eluting, have been widely applied in the clinic, and DESs decrease the incidence of ISR compared to bare metal stents (BMSs) mainly due to their inhibition to VSMCs proliferation [8–10]. A multi-center clinical control study conducted by Duda *et al.* [10] showed that after failure of the drug coating, a long-term risk of ISR remained, and there was no significant difference in patency rate between DESs and BMSs at 24 months. In addition, the migration of VSMCs could accelerate the directional movement of the proliferated VSMCs to the stent, promoting the intimal hyperplasia in the stent and increasing the incidence of stent restenosis [11]. Cypher and Taxus eluting stents can also inhibit the migration of VSMCs. Furthermore, Anemoside B4, FTY720 and other new drugs can inhibit the migration of VSMCs through different biological mechanisms [12, 13]. Therefore, it is much meaningful to find a novel material that can achieve both effects of promoting endothelialization and inhibiting long-term proliferation and migration of VSMCs.

Copper (Cu) is an essential trace metal element in the human body, which plays important roles in various catalytic reactions [14]. Early in 1980, McAuslan and Gole [15] found that Cu could stimulate the blood vessel formation. Subsequent studies demonstrated that Cu could not only stimulate the proliferation and migration of endothelial cells but also inhibit the proliferation of VSMCs and thrombosis formation, and furthermore, the Cu-bearing metals possessed strong antibacterial activity [16, 17].

Thus, a Cu-bearing 316L stainless steel (316L-Cu) was developed, which could reduce thrombosis formation by promoting proliferation and migration of endothelial cells and inhibiting both proliferation and migration of VSMCs, while also inhibiting the inflammation through continuous release of trace amount of Cu ions [18–20]. L605 alloy is a cobalt-based alloy that has been widely used as a stent platform for DESs, with superior strength than 316L stainless steel. Moreover, radiopacity of the stent was also improved due to the higher density of L605 alloy, and hence, the L605 stent can be delivered to lesion sites more safely and accurately. Furthermore, owing to the higher strength of L605 alloy, struts of the stent could be designed to be much thinner. Thinner struts can decrease the area contacting the vessel and thus reduce the metal coverage on vessel. Consequently, a novel type of L605 alloy with Cu addition (L605-Cu) has been developed, keeping the same microstructure, corrosion resistance and mechanical properties as the original L605 alloy and also accelerating the endothelialization [21]. Both effects of accelerating endothelialization and inhibiting VSMCs are of great importance for reducing ISR incidence. Nevertheless, there was no related study regarding the effect and mechanism of Cu-bearing metals on VSMCs.

In this study, both 316L-Cu stainless steel and L605-Cu alloy with varying Cu content were prepared. The effect of Cu-bearing metals on VSMCs was evaluated through *in vitro* test. In addition, the molecular level mechanism of Cu-bearing metals for inhibition of human aortic smooth muscle cells (HASMCs) was investigated. Furthermore, 316L-Cu stents were fabricated to study the influence of Cu-bearing metal on arterial neointimal hyperplasia through *in vivo* test. The findings from this study provide scientific evidence for clinical applications of Cu-bearing metals as promising stent materials.

## Materials and methods

### Material preparation

The chemical compositions of the experimental L605 and Cu-bearing L605 alloys (named as 2.0Cu, 2.5Cu and 3.5Cu) are listed in Table 1. We melted the alloys in a vacuum induction melting furnace and then forged them into 25 mm diameter bars. The bars were solution treated at 1200°C for 40 min, followed by water quenching. The chemical compositions of the experimental 316L and 316L-4.0Cu stainless steels are listed in Table 2. These steels were prepared by the same melting process as the above L605 alloys. After forging the steels were solution treated at 1100°C for 30 min, followed by water quenching, and then aged at 700°C for 6 h. Disk samples for experiments were cut from the above treated bars, with size of Φ10 × 2 mm. All the samples were ground using 400#, 600#, 800#, 1000#, 1500# and 2000# SiC papers successively before experiments, followed by washing in deionized water and absolute ethyl alcohol.

**Table 1.** Chemical compositions of experimental L605-xCu alloys (wt.%)

Materials	C	Si	Mn	P	S	Fe	Cr	W	Ni	Cu	Co
L605	0.066	0.51	1.08	0.002	0.002	1.0	19.00	14.7	11.3	–	Bal.
L605-2.0Cu	0.089	0.22	1.41	–	–	1.0	19.99	15.3	9.1	2.09	Bal.
L605-2.5Cu	0.053	0.36	0.73	0.002	0.002	1.0	19.96	14.9	10.4	2.49	Bal.
L605-3.5Cu	0.093	0.50	1.28	0.002	0.001	1.0	19.72	14.3	10.4	3.27	Bal.

**Table 2.** Chemical compositions of experimental 316L-xCu stainless steels (wt.%)

Materials	C	Si	Mo	P	S	Cr	N	Ni	Cu	Fe
316L	0.004	0.24	3.14	0.007	0.004	17.61	0.14	14.4	0.02	Bal.
316L-4.0Cu	0.016	0.22	3.02	0.005	0.001	18.18	0.14	14.5	4.36	Bal.

### Contact angle and surface properties

An angle meter (TBU-95 Data Physics, Germany) was used to measure the surface properties of L605-xCu alloys at room temperature. Water and bromonaphthalene (1 μl) were dropped onto the dried surfaces of disk samples using a microsyringe, respectively. The droplet was allowed to spread out across the surface for 1 min, and the water contact angles (WCAs) and the alpha-bromonaphthalene contact angles (BCAs) on the samples were recorded. The data were analyzed using the average value ± standard deviation for samples taken in triplicate. Besides, the surface tension (γ_s_^d^, γ_s_^p^) and polar components ratio (γ_s_^p^/γ_s_) were calculated using formula (1). The surface tension and corresponding components of the reagents used in the calculation are listed in Table 3. The contact angles of 316L and 316L-4.0Cu were detected with the same method as L605 series alloys.

(1) $$\left\{ \begin{array}{l} \gamma_{\text{L1}}\left(\text{1 }+\text{ cos}\theta_{\text{1}}\right)\;=\text{ 2}{\left(\gamma_{\text{L1}}{\;}^{\text{d}}\gamma_{\text{s}}{\;}^{\text{d}}\right)}^{\text{1}/\text{2}}+\text{2}{\left(\gamma_{\text{L1}}{\;}^{\text{p}}\gamma_{\text{s}}{\;}^{\text{p}}\right)}^{\text{1}/\text{2}}\\ \gamma_{\text{L2}}\left(\text{1 }+\text{ cos}\theta_{\text{2}}\right)\;=\text{ 2}{\left(\gamma_{\text{L2}}{\;}^{\text{d}}\gamma_{\text{s}}{\;}^{\text{d}}\right)}^{\text{1}/\text{2}}+\text{2}{\left(\gamma_{\text{L2}}{\;}^{\text{p}}\gamma_{\text{s}}{\;}^{\text{p}}\right)}^{\text{1}/\text{2}} \end{array}\right.$$

**Table 3.** Surface tension (r_s_) and components ($r_{s}^{p}$, $r_{s}^{d}$) of deionized water and alpha-brominated naphthalene, respectively

Reagents	γ_s_^p^	γ_s_^d^	γ_s_
Deionized water	51.0	21.8	72.8
α-bromonaphthalene	0	44.6	44.6

### Cu ion release

Disk samples of 316L-xCu (*x* = 0, 4.0) and L605-xCu (*x* = 0, 2.0, 2.5, 3.5) were immersed in a 0.9% NaCl solution at a ratio of 1.25 cm^2^/ml for 1, 7, 14 and 28 days, respectively. The immersion medium was then analyzed using a graphite furnace atomic absorption spectrophotometer (Hitachi Z-2300, Japan).

### Cell culture

The HASMCs (KeyGen, China) were cultured with Dulbecco’s modified eagle medium (DMEM, HyClone, USA), supplemented with 10% fetal bovine serum (Gibco, Life Technologies, USA), 80 U/ml penicillin and 0.08 mg/ml streptomycin, according to the supplier’s recommendations. A humidified atmosphere of 5% CO_2_ and 95% air was used for incubating cells. After reaching 80% confluence, cells were passaged using 0.25 wt.% trypsin-EDTA solution (Gibco, Life Technologies, USA). The following experiments were conducted with cells at Passages 4–6.

### Preparation of extracts

In accordance with ISO 10993-5, disk samples of 316L-xCu (*x* = 0, 4.0) and L605-xCu (*x* = 0, 2.0, 2.5, 3.5) were incubated in serum-free DMEM medium with 1.25 cm^2^/ml. The culture media used were collected as extracts after the 72-h incubation at 37°C.

### CCK-8 assay

Cell counting kit 8 (CCK-8, Dojindo, Japan) was used to determine the cytotoxicity of various Cu-bearing metals on HASMCs. Disk samples were placed into 48-well plates prior to adding a suspension of 4 × 10^4^ cells onto the samples. After co-culture for 24 or 72 h, the solution was replaced with a 500-μl mixture of fresh medium (450 μl) and CCK-8 solution (50 μl), followed by incubation at 37°C for 4 h. Then, 200 μl of liquid from each well was transferred to a fresh 96-well plate. The absorbance was measured at 450 nm using a microplate reader.

### Dynamic clotting test

Healthy volunteers provided blood for the study. First, 100 μl of blood with anticoagulant (blood/anticoagulant = 9/1, where the anticoagulant was 2.5% sodium citrate solution) was dropped onto the surfaces of samples, and 10 μl of 0.2 mol/l CaCl_2_ was simultaneously added. At setting times of 10, 30, 50, 70 and 90 min, rinsing the samples with distilled water and collecting the rinsed water were both done. Next, 200 μl of rinsed water was transferred to a fresh 96-well plate. The absorbance of free hemoglobin remaining in the fluid at λ = 545 nm was measured by a microplate reader.

### Hemolysis test

An anticoagulated sample of fresh whole blood was obtained legally from a healthy volunteer. ISO 10993-4:2009 and ASTMF756-00 were followed for the hemolysis experiments. An initial volume of anticoagulant blood of 4 ml was diluted with a solution of 0.9% sodium chloride of 5 ml. A solution containing 0.9% NaCl was also included in each sample. The negative control solution was prepared using 262 μl of 0.9% NaCl solution (*n* = 3), while the positive control was prepared using 262 μl of double distilled water (*n* = 3). The samples and controls were incubated at 37°C for 30 min, and then followed by a 60-min incubation with anticoagulant blood and centrifugation at 3000 rpm for 5 min. Following that, a microplate reader (Thermo, USA) was used to measure the absorbance at 545 nm of the supernatant. Following is the formula used to calculate the hemolysis ratio:

(2) $$R=\;(S-C_{\text{1}})/(C_{\text{2}}-C_{\text{1}})\times \text{1}00\%$$

There are four variables in this equation: *R* stands for the hemolysis ratio (%), *S* for the absorbance of the sample (%), *C*_1_ for the absorbance of the negative control (%) and *C*_2_ for the absorbance of the positive control (%).

### Transwell assay

Transwell assays were used to assess the effect of different ionic extracts on cell migration. After suspension at 4 × 10^5^ cells/ml with the extract, 200 μl of suspended HASMCs were added to an upper chamber of 8 μm diameter, and 600 μl of extract supplemented with 20% serum were placed in the lower chamber. Cotton swabs were used to gently remove the cells in the upper chamber of the insert after washing with PBS for 12 h. Fixing and staining of upper chamber cells with 95% alcohol and 0.1% crystal violet were used for dyeing. A total of five views were selected randomly under a microscope, and the average number of cells was calculated to determine how many cells migrated.

### Wound scratch assay

The cell scratch test was used to examine the effect of different material extracts on the ability of HASMCs to laterally migrate. First, the concentration of HASMCs was adjusted to 3 × 10^5^ cells/ml, and 2 ml of cell suspension was added into each pore of the six-well plates. Then, cells were cultured until they covered 90% of the pore. A vertical scratch line was created using a standard 10-µl pipette tip at the middle of the well. The liquid in the hole was aspirated and slowly rinsed with PBS, and then 2 ml of serum-free extract was added to each well. Before the extraction solution was added and after added for 24 h, respectively, photos were taken at the same location. Photoshop and Image J software were used to calculate the area of scratches in the two groups of photos before and after scratching to quantify the migration area.

### 5-ethynyl-2′-deoxyuridine assay

Disk samples were placed in 48-well plates. Then, the HASMC suspension was placed onto each disk (1 × 10^5^ cells/well), which was cultured to the normal growth stage. After 24 h, 5-ethynyl-2′-deoxyuridine (EdU) was added to the HASMCs, which was cultured at 37°C for 2 h. Subsequently, the cells were stained using the Click-iT EdU-594 (Beyotime Biotechnology, China).

### Isolation of mRNA and quantitative real-time polymerase chain reaction

A total of 4 × 10^4^ cells were co-cultured with the samples for 72 h. Total RNA was isolated from cells using RNAiso Plus (TAKARA Biotechnology, China). A total volume of 20 µl of RNA (1 µg) was reverse-transcribed using a PrimeScript RT reagent kit (Perfect Real Time) (TAKARA Biotechnology, China). With a volume of 20 µl, quantitative real-time PCR was conducted on cDNA samples using the LightCycler480 II instrument (Roche, Basel, Switzerland) with the SYBR Green Premix Ex Taq II (TAKARA Biotechnology, China). Following is the sequence of primers: *Fgf2*-F, 5′-AGGAGTGTGTGCTAACCGTT-3′; *Fgf2*-R, 5′-CAGTTCGTTTCAGTGCCACA-3′; *Pdgfb*-F, 5′-TTGTGCGGAAGAAGCCAATC-3′; *Pdgfb*-R, 5′- GAATGGTCACCCGAGTTTGG-3′; *Hgf*-F, 5′- AAACAATGCCTCTGGTTCCC-3′; *Hgf*-R, 5′-ACTCCAGGGCTGACATTTGA-3′; *Nrf2*-F, GTGGCATCACCAGAACACTCAG; *Nrf2*-R, TGACACTTCCAGGGGCACTATC; *GCLC*-F, CATTGATTGTCGCTGGGGAG; and *GCLC*-R, CTGGGCCAGGAGATGATCAA; *GCLM*-F, TGTATCAGTGGGCACAGGTA; *GCLM*-R, GTGCGCTTGAATGTCAGGAA; *NQO1*-F, ACGCCCGAATTCAAATCCTG; *NQO1*-R, GTCAGTTGGGATGGACTTGC; *HO1*-F, CGTTCCTGCTCAACATCCAG; *HO1*-R, TGAGTGTAAGGACCCATCGG; *GAPDH*-F, 5′-TCAAGAAGGTGGTGAAGCAGG-3′; and *GAPDH*-R, 5′-TCAAAGGTGGAGGAGTGGGT-3′. An internal control was performed by measuring the expression level of glyceraldehyde-3-phosphate dehydrogenase (GAPDH). Relative expression was evaluated using the 2^-ΔΔCT^ method as previously described [22].

### Western blotting

The samples were co-cultured with 4 × 10^4^ cells for 72 h. Cells were pretreated with 8 µg/ml AKT activator SC79 (Beyotime Biotechnology, China), or 25 µM Nrf2 inducer tertiary butylhydroquinone (TBHQ) (Med Chem Express, USA) for 24 h. A M-PER mammalian protein extraction reagent (Thermo Scientific, USA) supplemented with Halt Protease and Phosphatase Inhibitor Cocktail (Thermo Scientific, USA) was used to extract all the proteins from the cells. Western blot analysis was carried out on the supernatants after debris was removed via centrifugation at 800 rpm for 15 min at 4°C. There were 30 µg of protein loaded and separated onto 4-12 gradient minigels (Thermo Fisher, Waltham, USA) and electrotransferred onto polyvinylidene difluoride (PVDF) membranes (Thermo Fisher). Blocking the membranes with 5% milk or 5% BSA powder (BD Bioscience, CA), washing with Tris-Buffered Saline containing Tween 20 (TBST) (Sigma-Aldrich) and incubating overnight at 4°C with rabbit polyclonal anti-Ser473-phosphorylated-AKT antibody (1:1000, Cell Signaling), rabbit polyclonal anti-AKT antibody (1:1000, Abcam, USA), rabbit polyclonal anti-NRF2 antibody (1:1000, Proteintech), GCLC polyclonal antibody (1:1000, Proteintech), GCLM polyclonal antibody (1:1000, Proteintech), rabbit polyclonal anti-NQO1 antibody (1:1000, Abcam), HO-1/HMOX1 polyclonal antibody (1:1000, Proteintech) and GAPDH polyclonal antibody (1:5000, Proteintech). After washing with TBST, the membranes were incubated with peroxidase-conjugated rabbit IgG (Santa Cruz Biotechnology, CA) as the secondary antibody. The membranes were developed using Thermo Scientific’s SuperSignal West Femto Maximum Sensitivity Substrates (Thermo Scientific, USA) after being washed once more with TBST. Image Lab 4.1 (BioRad, Hercules, CA) was used to image and analyze protein signals.

### Stent manufacture and stent implantation procedure

**Figure 1.**
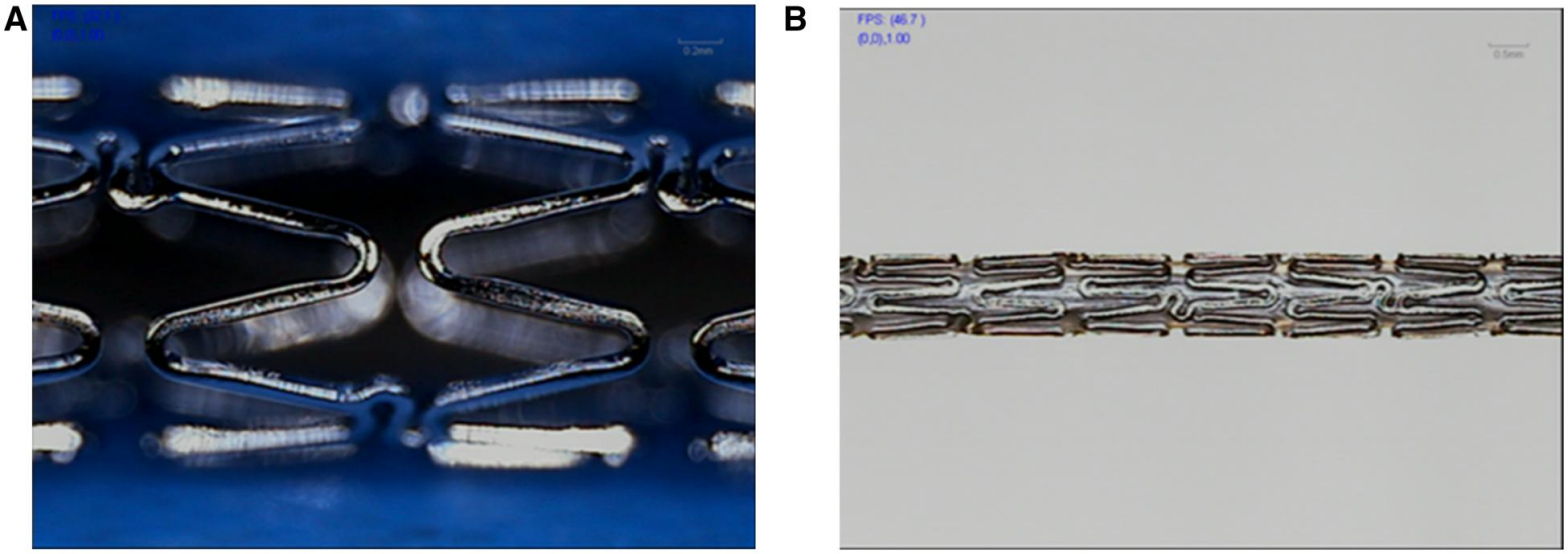
Photos of the stent (**A**) and the delivery system of the stent (**B**).

316L and 316L-4.0Cu vascular stents were manufactured on the material mini-tubes by a laser cutting machine (HT-1330 cutting machine of Kunshan Situo Machine Co., LTD), followed by an electrochemical polishing. The axial length of the stent was 19.6 mm, and the strut thickness was ∼90 μm. It was necessary for the stent to obtain a smooth surface through electrochemical polishing to remove the defects on its surface. The original stent configuration and the configuration after crimping are shown in (Figure 1A and B), respectively, which were used for the animal experiment.

Six male New Zealand white rabbits (2.5–3.0 kg) were randomly divided into 316L-4.0Cu group and 316L group, with three rabbits in each group. And 100 mg aspirin (Bayer, Germany) and 75 mg clopidogrel (Sanofi, French) were given 3 days before the implantation. The rabbits were anesthetized by an intramuscular injection of ketamine (3.0 mg/kg) and 2 ml 3% sodium pentobarbital. The animals were placed in the supine position and immobilized. Left neck was shaved and sterilized with povidone-iodine. Onwards, left carotid artery was punctured with Seldinger method. Then, 5F sheath was inserted with the injection of heparin (200 U/kg). A 5F Cobra catheter was placed at the end of the abdominal aorta under the guidance of a guide wire of 0.035 inch in diameter for iliac artery angiography. Stent size was selected based on the diameter of the target vessel (stent expanded diameter: vascular diameter = 1.5:1). A guide wire (0.014 inch) was placed in the left iliac artery. Stents were pushed along guide wires to the target vessel site and released with 6–8 atm single expansion for 3–5 s. A repeat angiography was performed to ensure that there had been no dissections or thromboses. The balloon, guide wire and sheath were removed, the left carotid artery was ligated, the incision was sutured, and the surgery was completed. Postoperative intramuscular injection of 8 × 10^5^ U penicillin sodium/each rabbit was made for three days to prevent infection. Aspirin (100 mg/day) and clopidogrel (75 mg/day) were then given until the animal was sacrificed.

### Histopathological evaluation

Six months after stent implantation, all the experimental animals were euthanized with sodium pentobarbital injection and the treated artery segments were collected. The artery was perfused with heparin saline, separated rapidly and fixed with a 10% formaldehyde solution. The samples with stent were embedded by methyl methacrylate and sliced for 50 μm in thickness with a hard tissue microtome (LEICA, Germany). All the sections were stained with HE, and then the sections were transferred to two pathologists for pathology evaluation. The score for VSMCs proliferation was determined as the local coverage of the stent by proliferating VSMCs and graded from 1 to 3 (1 = 0–25%, 2 = 25–75%, 3 = 75–100%).

### Statistical analysis

The mean ± standard deviation (SD) was used to express all the data. Data from *in vitro* experiments were analyzed using 1-way ANOVA, followed by a Bonferroni post-test (GraphPad Prism 9, USA). Statistical significance between the two groups was analyzed by the unpaired *t* test. The value of *P *<* *0.05 was considered statistically significant.

## Results

### Surface properties of materials

**Figure 2.**
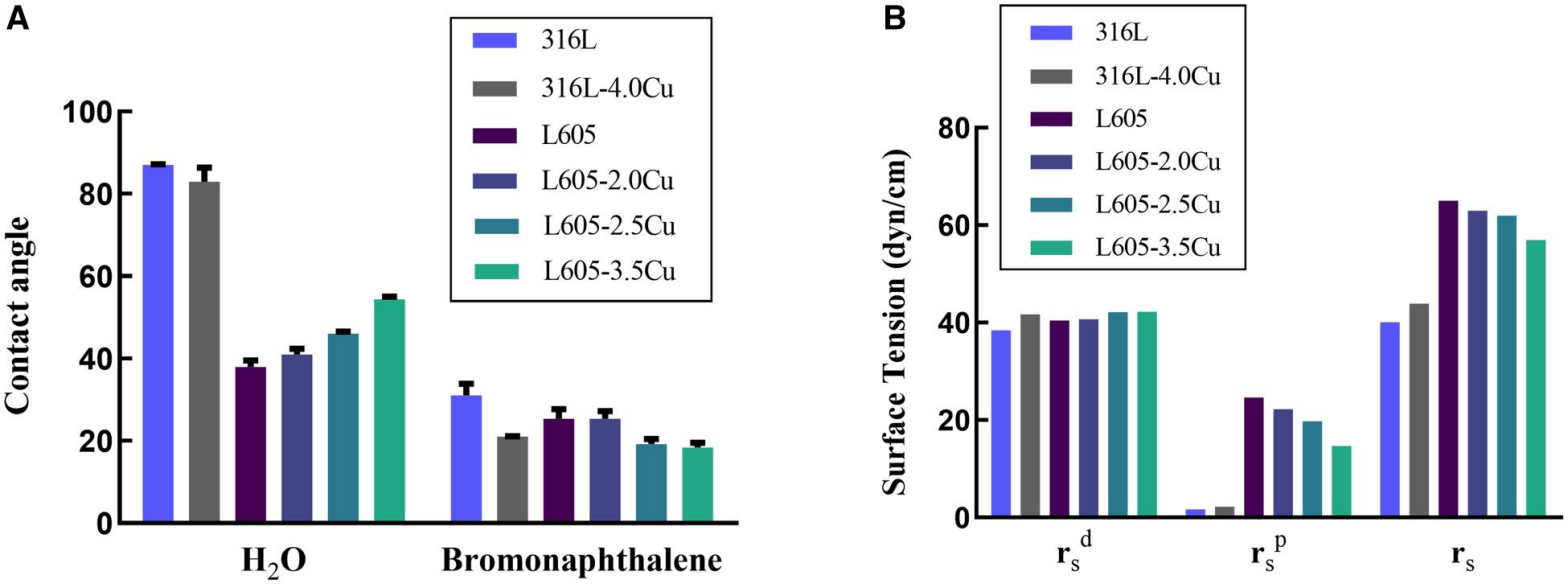
(**A**) Contact angles of L605-xCu alloy and 316L-xCu stainless steel; (**B**) surface tension of L605-xCu alloy and 316L-xCu stainless steel.

The WCA and BCA of L605-xCu alloy and 316L-xCu stainless steel samples are shown in Figure 2A. The WCAs of the samples increased with increasing the Cu content for L605-xCu alloy, but there was no significant difference between 316L and 316L-4.0Cu stainless steels. However, the regularity of the BCAs of the samples decreased with the Cu content. Additionally, the surface tension (r_s_) and corresponding components (r_s_^p^, r_s_^d^) were calculated, as shown in Figure 2B. As the Cu content was increased, the dispersion force (r_s_^d^) of the samples remained almost unchanged, while the polar force (r_s_^p^) of the samples was reduced. Moreover, the surface tension of all the samples decreased. According to the above results, the polar component ratio (r_s_^p^/r_s_) decreased as the Cu content was increased for both materials.

### Cu ion release, cytotoxicity and blood compatibility

**Figure 3.**
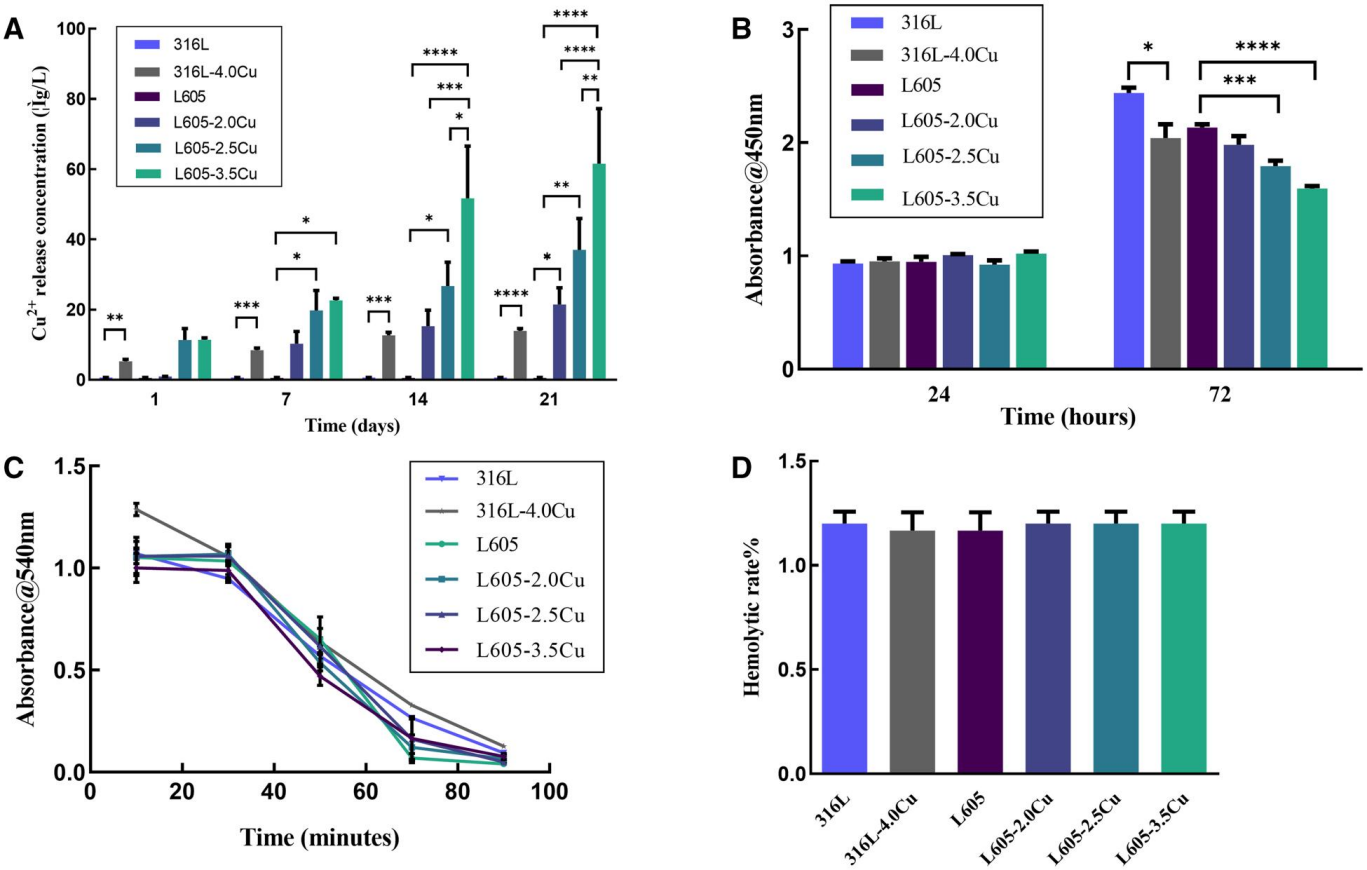
(**A**) Cumulative concentration of Cu ions released from Cu-bearing 316L and Cu-bearing L605 samples. (**B**) CCK-8 cytotoxic assay of different Cu-bearing 316L and Cu-bearing L605 samples to HASMCs after 24 h and 72 h co-cultures. Blood compatibility test results of different Cu-bearing 316L and Cu-bearing L605 samples: (**C**) clotting time curves. (**D**) hemolysis rates. Each value was the mean ± standard deviation of triplicate determinations. (*P < 0.05, **P < 0.01, ***P < 0.001 and ****P < 0.0001).

As shown in Figure 3A, the Cu ion concentration released from 316L-xCu stainless steel and L605-xCu alloy after 1, 7, 14 and 21 days immersions. After immersion for 1 day, there were fewer Cu ions detected for the L605-2.0Cu sample; however, there were up to >10 µg/l of Cu ions for both L605-2.5Cu and L605-3.5Cu samples. There was no significant difference among the groups after 1 day of immersion. The trend for concentration of released Cu ions increased as the immersion time was increased. Starting from 7 days of immersion, significantly larger amounts of Cu ions were released from the L605-2.5Cu and L605-3.5Cu samples than the L605 sample. At 21 days of immersion, the Cu ions released from L605-2.0Cu sample were significantly higher than those from L605 sample. At 14 and 21 days of immersions, the Cu ions released from L605-3.5Cu sample were significantly greater than those from L605-2.0 Cu and L605-2.5Cu. (****P < 0.0001; ***P < 0.001; **P < 0.01 and *P < 0.05). With the same tendency, the trend for concentration of Cu ions released from 316L-xCu stainless steel increased as the immersion time was increased. However, the Cu ions concentration released from 316L-4.0Cu sample was lower than that from L605-3.5Cu sample, even lower than L605-2.0Cu alloy.

CCK-8 was performed to compare the cytotoxicity and cell proliferation of different Cu-bearing 316L and Cu-bearing L605 samples to HASMCs after 24 and 72 h co-cultures. It can be seen from Figure 3B that cells in all the groups grew over time and showed non-cytotoxicity. Compared to 316L, 316L-4.0Cu had a significant inhibitory effect on HASMCs proliferation at 72 h, the absorbance@450nm for 316L and 316L-4.0Cu groups were 2.44 ± 0.08 and 2.04 ± 0.21 at 72 h, respectively. The L605-xCu showed significant inhibitory effect on cell proliferation compared to L605 at 72 h, the absorbance@450nm for L605, L605-2.0Cu, L605-2.5Cu and L605-3.5Cu groups were 2.13 ± 0.06, 1.98 ± 0.15, 1.79 ± 0.10 and 1.60 ± 0.04 at 72 h, respectively.

The dynamic clotting time reflects the degree of activation of endogenous clotting factors after a material contacts blood, which can be used to evaluate the effect of materials on endogenous clotting. The higher the absorbance value, the lower the activation degree of the coagulation factor, and the better the anticoagulant property of the material. As shown in Figure 3C, the anticoagulant properties of different Cu-bearing 316L and L605 samples showed no significant difference; however, some tendencies could still be found. During the first 60 min co-culture with whole blood, the more the Cu added in the alloy, the lower the absorbance value was. Over 60 min co-culture with whole blood, the more the Cu added, the higher the absorbance value was.

If the hemolysis rate of a material is <5%, the material meets the hemolysis requirement for the medical materials. It can be seen that the hemolysis rates of Cu-bearing metals (316L-4.0Cu and L605-Cu) were all <1.5% (Figure 3D). Furthermore, there was no statistically significant difference in hemolysis rate between 316L and 316L-4.0Cu, as well as L605 and L605-Cu.

In summary, the above experiments demonstrated that the release of Cu ions from Cu-bearing 316L and Cu-bearing L605 samples significantly increased with the immersion time and the Cu content. 316L, L605 and L605-2.0Cu had negligible cytotoxic effects on HASMCs for 24 h co-culture. 316L-4.0Cu, L605-2.5Cu and L605-3.5Cu could inhibit HASMCs proliferation for 72 h co-culture. Thus, the Cu-bearing metals showed good blood compatibility.

### Migration and proliferation of HASMCs

**Figure 4.**
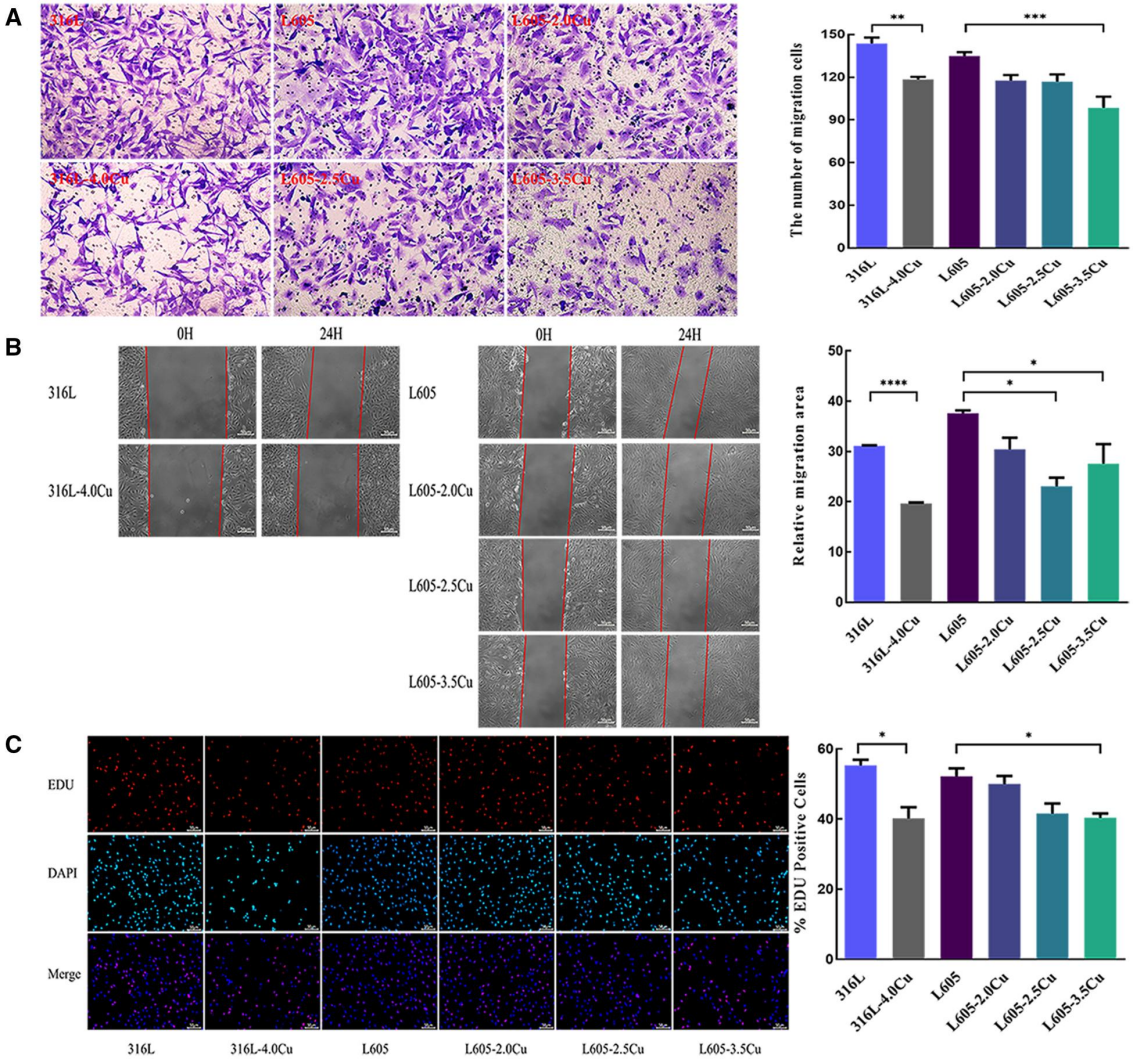
Cells migration and proliferation induced by Cu-bearing 316L and Cu-bearing L605 samples: (**A**) images and quantitative analysis of HASMCs stained with 0.1% crystal violet in transwell assays (200×); (**B**) images and quantitative analysis of HASMCs in the wound scratch assay (100×); (**C**) images of HASMCs seeded on 316L, 316L-4.0Cu, L605 and L605-Cu in EdU assay and percentages of EdU-positive cells on different materials (100×).

As shown in Figure 4A, it appears that fewer cells moved through the membrane due to the effect of 316L-4.0Cu and L605-Cu extracts, revealing that both 316L-4.0Cu and L605-Cu could suppress the migration of HASMCs. The numbers of migrated cells in the 316L, 316L-4.0Cu, L605, L605-2.0Cu, L605-2.5Cu and L605-3.5Cu groups were 143.60 ± 9.40, 118.40 ± 3.85, 134.80 ± 2.75, 117.60 ± 3.86, 116.80 ± 5.08 and 98.40 ± 7.87, respectively. Moreover, there was a statistically significant difference in quantitative data between 316L and 316L-4.0Cu groups, as well as between L605 and L605-3.5Cu groups.

The results of the scratch assay showed that the cell migration areas of 316L-4.0Cu and L605-Cu groups significantly decreased compared with 316L and L605, respectively (Figure 4B). The relative migration areas (%) of 316L, 316L-4.0Cu, L605, L605-2.0Cu, L605-2.5Cu and L605-3.5Cu groups were 31.09 ± 0.21, 19.66 ± 0.36, 37.60 ± 0.98, 30.42 ± 4.02, 23.09 ± 2.97 and 27.55 ± 6.79, respectively. The relative migration areas of L605-2.5Cu and L605-3.5Cu groups were significantly smaller than that of L605 group, and the relative migration areas of 316L-4.0Cu group was significantly smaller than that of 316L group. This result suggests that Cu-bearing metals could inhibit the migration of HASMCs.

The findings from the EdU assay implied that the surfaces of both 316L-4.0Cu and L605-Cu might suppress the proliferation of HASMCs. As shown in Figure 4C, new proliferative cells were stained red, while all nuclei, including proliferating cells, were stained blue. The EdU-positive cells (%) of 316L, 316L-4.0Cu, L605, L605-2.0Cu, L605-2.5Cu and L605-3.5Cu groups were 55.31 ± 2.73, 40.20 ± 5.45, 40.27 ± 5.92, 35.19 ± 3.10, 34.95 ± 4.48 and 30.04 ± 3.08, respectively. There was a statistically significant difference in new proliferative cells between L605 and L605-3.5Cu, as well as between 316L and 316L-4.0Cu.

### Gene expressions

**Figure 5.**
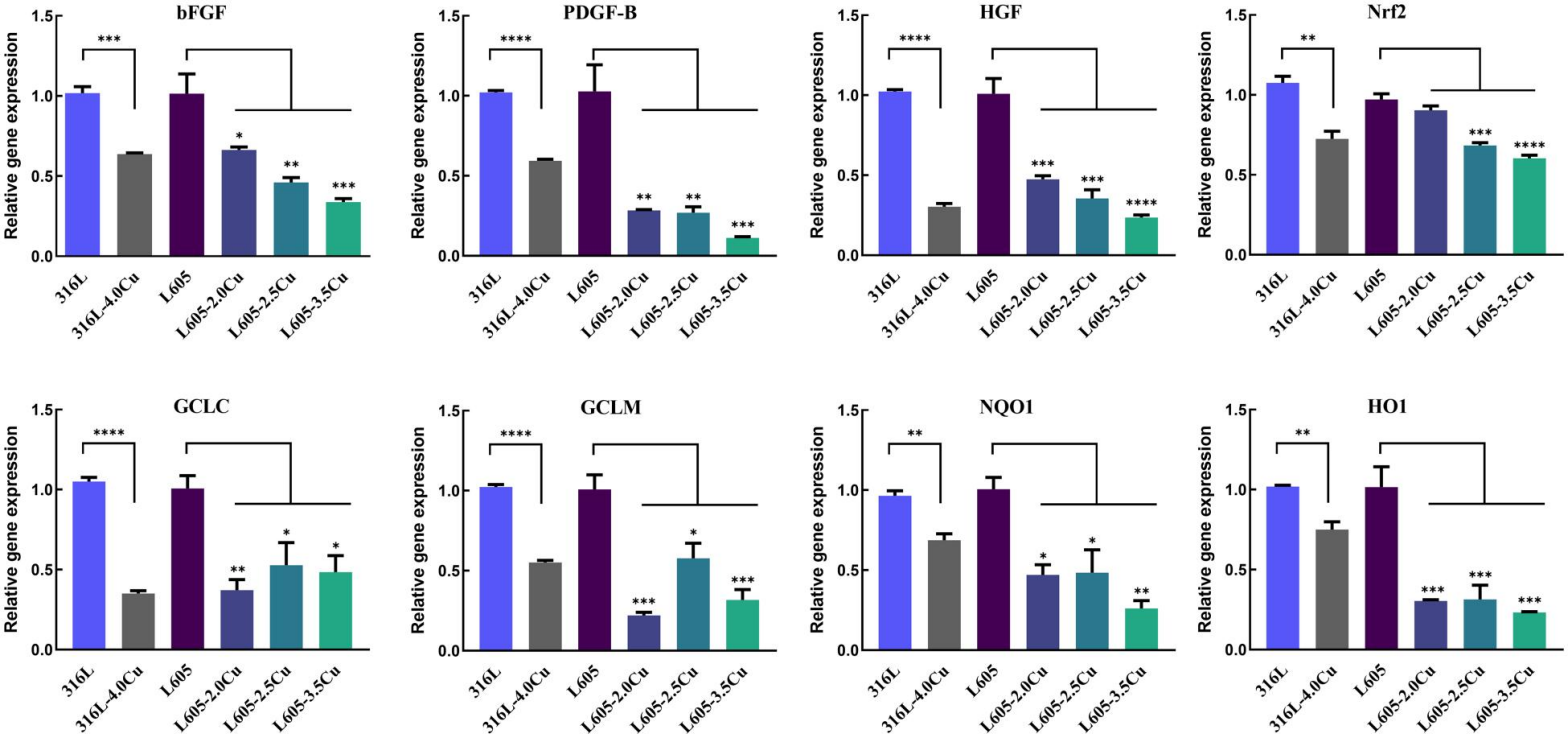
Relative mRNA expressions of *bFGF*, *PDGF-B*, *HGF*, *Nrf2*, *GCLC*, *GCLM*, *NQO1* and *HO1* in HASMCs seeded on 316L, 316L-4.0Cu, L605, L605-2.0Cu, L605-2.5Cu and L605-3.5Cu, in comparison with 316L and L605, respectively. *P <* *0.05, **P <* *0.01, ***P <* *0.001.

The effect of 316L-Cu and L605-Cu alloy on the mRNA expression for various factors was examined by real-time PCR analysis, as shown in Figure 5. *bFGF* plays a pivotal role in the migration of HASMCs, and the relative mRNA expressions of *bFGF* in HASMCs were suppressed by 0.664 ± 0.015, 0.460 ± 0.061 and 0.338 ± 0.047 folds in L605-2.0Cu, L605-2.5Cu and L605-3.5Cu groups, respectively, in comparison with the L605 group. Compared with 316L group, the expressions of *bFGF* in HASMCs were suppressed by 0.637 ± 0.013 folds in 316L-4.0Cu group. *PDGF-B* is a potent mitogenic factor for VSMCs. After cultured on the surface of samples for 3 days, the relative mRNA expressions of *PDGF-B* in HASMCs were suppressed by 0.283 ± 0.008, 0.270 ± 0.038 and 0.113 ± 0.012 folds in L605-2.0Cu, L605-2.5Cu and L605-3.5Cu groups, respectively, in comparison with the L605 group. Compared with 316L group, the expressions of *PDGF-B* in HASMCs were suppressed by 0.593 ± 0.017 folds in 316L-4.0Cu group. *HGF* triggers signaling cascades to mediate the migration of VSMCs. After cultured on the surface of samples for 3 days, the relative mRNA expressions of *HGF* in HASMCs were suppressed by 0.474 ± 0.019, 0.355 ± 0.130 and 0.236 ± 0.032 folds in L605-2.0Cu, L605-2.5Cu and L605-3.5Cu groups, respectively, in comparison with the L605 group. And compared with 316L group, the expressions of *HGF* in HASMCs were suppressed by 0.303 ± 0.036 folds in 316L-4.0Cu group.

*Nrf2* plays an important role in protecting against atherosclerosis. Downregulation of *Nrf2* could suppress the migration and proliferation of VSMCs in atherosclerosis disease [23]. After cultured on the surfaces of 316L-4.0Cu and L605-Cu samples for 3 days, the relative mRNA expressions of *Nrf2* in HASMCs were suppressed by 0.904 ± 0.047, 0.684 ± 0.031 and 0.604 ± 0.035 folds in L605-2.0Cu, L605-2.5Cu and L605-3.5Cu groups, respectively, in comparison with the L605 group. Compared with 316L group, the expressions of *Nrf2* in HASMCs were suppressed by 0.725 ± 0.083 folds in 316L-4.0Cu group. *HO-1*, *NQO1*, *GCLC* and *GCLM* are downstream genes that can be activated by *Nrf2*. After cultured on the surfaces of samples for 3 days, the relative mRNA expressions of *HO-1* in HASMCs were suppressed by 0.305 ± 0.013, 0.314 ± 0.153 and 0.233 ± 0.007 folds; the relative mRNA expressions of *NQO1* were suppressed by 0.470 ± 0.110, 0.484 ± 0.247 and 0.261 ± 0.083 folds; *GCLC* was suppressed by 0.372 ± 0.114, 0.528 ± 0.243 and 0.485 ± 0.178 folds; and *GCLM* was suppressed by 0.221 ± 0.034, 0.577 ± 0.163 and 0.317 ± 0.113 folds in L605-2.0Cu, L605-2.5Cu and L605-3.5Cu groups, respectively, in comparison with the L605 group. Compared with the 316L group, the relative mRNA expressions of *HO-1*, *NQO1*, *GCLC* and *GCLM* were suppressed by 0.750 ± 0.084, 0.687 ± 0.069, 0.351 ± 0.030, 0.551 ± 0.024 folds in 316L-4.0Cu group, respectively. Thus, the Cu-bearing metals, 316L-4.0Cu and L605-Cu, could significantly inhibit the gene expressions of *bFGF*, *PDGF-B*, *HGF*, *Nrf2* and *Nrf2*-related downstream genes in HASMCs.

### Western blotting

**Figure 6.**
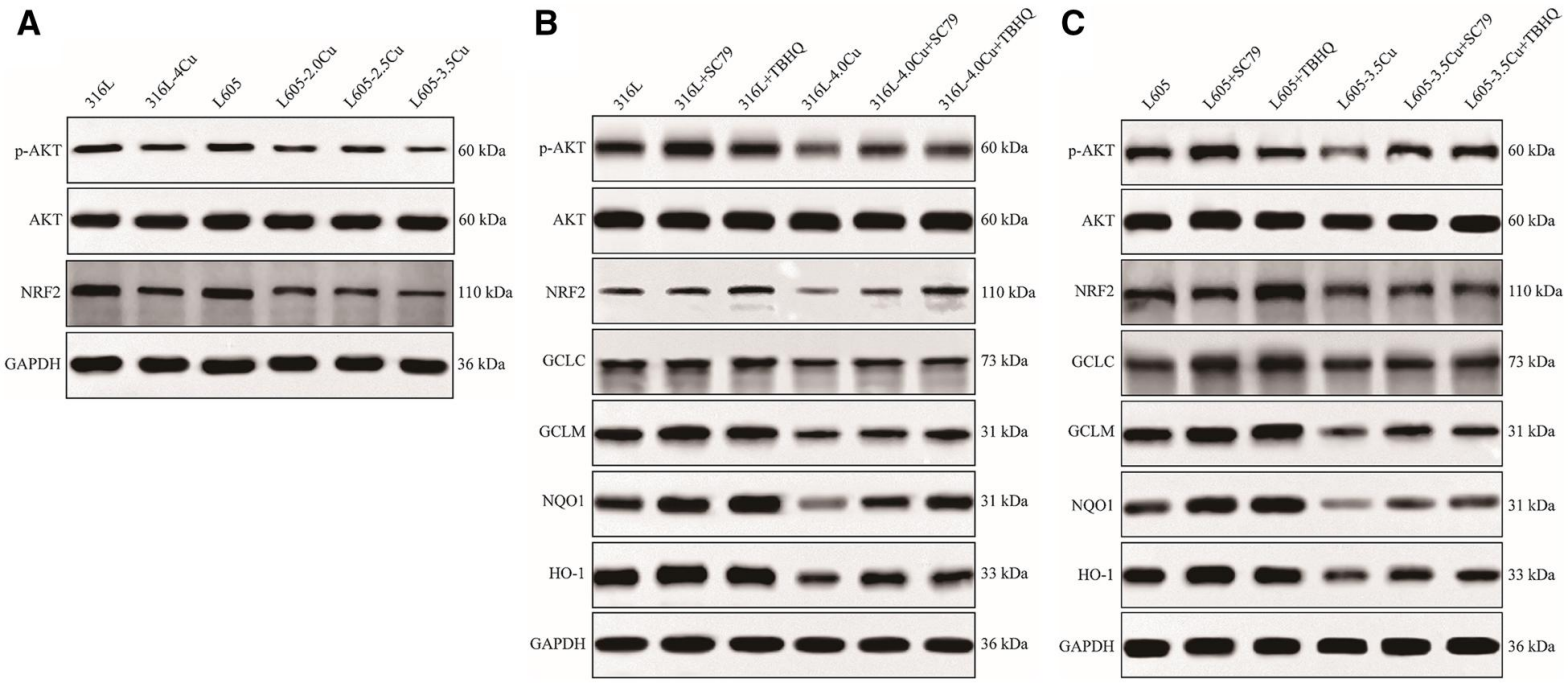
(**A**) Relative p-AKT/AKT and Nrf2 protein expressions in HASMCs seeded on 316L, 316L-4.0Cu, L605 and L605-Cu; (**B**) expressions of phospho-AKT, Nrf2, HO-1, NQO1, GCLC and GCLM in HASMCs seeded on 316L and 316L-4.0Cu; (**C**) expressions of phospho-AKT, Nrf2, HO-1, NQO1, GCLC and GCLM in HASMCs seeded on L605 and L605-3.5Cu.

AKT signaling pathways have been demonstrated to be involved in a number of cellular activities such as cell division, migration and differentiation in previous studies [24, 25]. AKT is a downstream pathway of PI3K and is regulated by PI3K [26]. Wang *et al*. [27] found that the P13K inhibitor exposure reduced the Nrf2 activity in ARPE cells. Nrf2 may play a role in preventing atherosclerosis through its suppression of migration, proliferation and inflammation of VSMCs [28]. As shown in Figure 6A, the expressions of phosphorylated(p)-Akt and Nrf2 were detected in HASMCs cultured with 316L-Cu and L605-Cu samples by western blotting. Both phosphorylated(p)-Akt and Nrf2 expressions were found to significantly decrease in 316L-Cu and L605-Cu, compared with 316L and L605, respectively. Also, in the L605-Cu group, the level of phosphorylated(p)-Akt and Nrf2 continuously decreased as the Cu content increased. These results indicated that both 316L-Cu and L605-Cu could inhibit the proliferation and migration of HASMCs by decreasing the phosphorylation of AKT and the expression of Nrf2 protein.

Furthermore, to investigate the possibility that the AKT/Nrf2/ARE signaling pathway might be involved in the inhibition of migration and proliferation of VSMCs by Cu-bearing metals, SC79 (an activator of AKT) and TBHQ (an inducer of Nrf2) were used to activate the AKT/Nrf2 signaling pathway. As shown in Figure 6B, 316L-4.0 Cu significantly decreased the expressions of phospho-AKT, Nrf2 and GCLM in HASMCs compared with 316L. SC79 treatment could significantly increase the expressions of phospho-AKT and GCLM in 316L group, and also significantly increase the expressions of GCLC and NQO1 in 316L-4.0 Cu group. After treatment with SC79, GCLC, NQO1 and HO-1 expressions tended to increase in 316L group, and the expressions of phospho-AKT, Nrf2, GCLM and HO-1 tended to increase in 316L-4.0 Cu group. Moreover, TBHQ treatment could significantly increase the expressions of Nrf2, GCLM and NQO1, except phospho-AKT in 316L group. TBHQ treatment could greatly increase the expressions of Nrf2 and NQO1 proteins in 316L-4.0 Cu group. After treatment with TBHQ, GCLC, GCLM and HO-1 expressions tended to increase in 316L-4.0 Cu group.

As shown in Figure 6C, L605-3.5Cu significantly decreased the expressions of phospho-AKT, Nrf2 and HO-1, NQO1, GCLC and GCLM in HASMCs, compared with L605, respectively. SC79 treatment could significantly increase the expressions of phospho-AKT, GCLC, GCLM, NQO1 and HO-1 in L605 group, and also significantly increase the expressions of phospho-AKT, GCLC, NQO1 and HO-1 in L605-3.5Cu group. After treatment with SC79, Nrf2 expression tended to increase in both L605 groups and L605-3.5Cu groups, and GCLM expression tended to increase in L605-3.5Cu group. Moreover, TBHQ treatment could significantly increase the expressions of Nrf2, GCLC, GCLM, NQO1 and HO-1, except phospho-AKT in L605 group, and TBHQ treatment could significantly increase the expressions of Nrf2, GCLC, NQO1 and HO-1, except phospho-AKT in L605-3.5Cu group. After treatment with TBHQ, GCLM expression tended to increase in L605-3.5Cu group.

These results showed that both 316L-Cu and L605-Cu inhibited the proliferation and migration of HASMCs through the AKT/Nrf2/ARE pathway, and L605-Cu showed better biological reaction.

### Angiography of stent implantation

**Figure 7.**
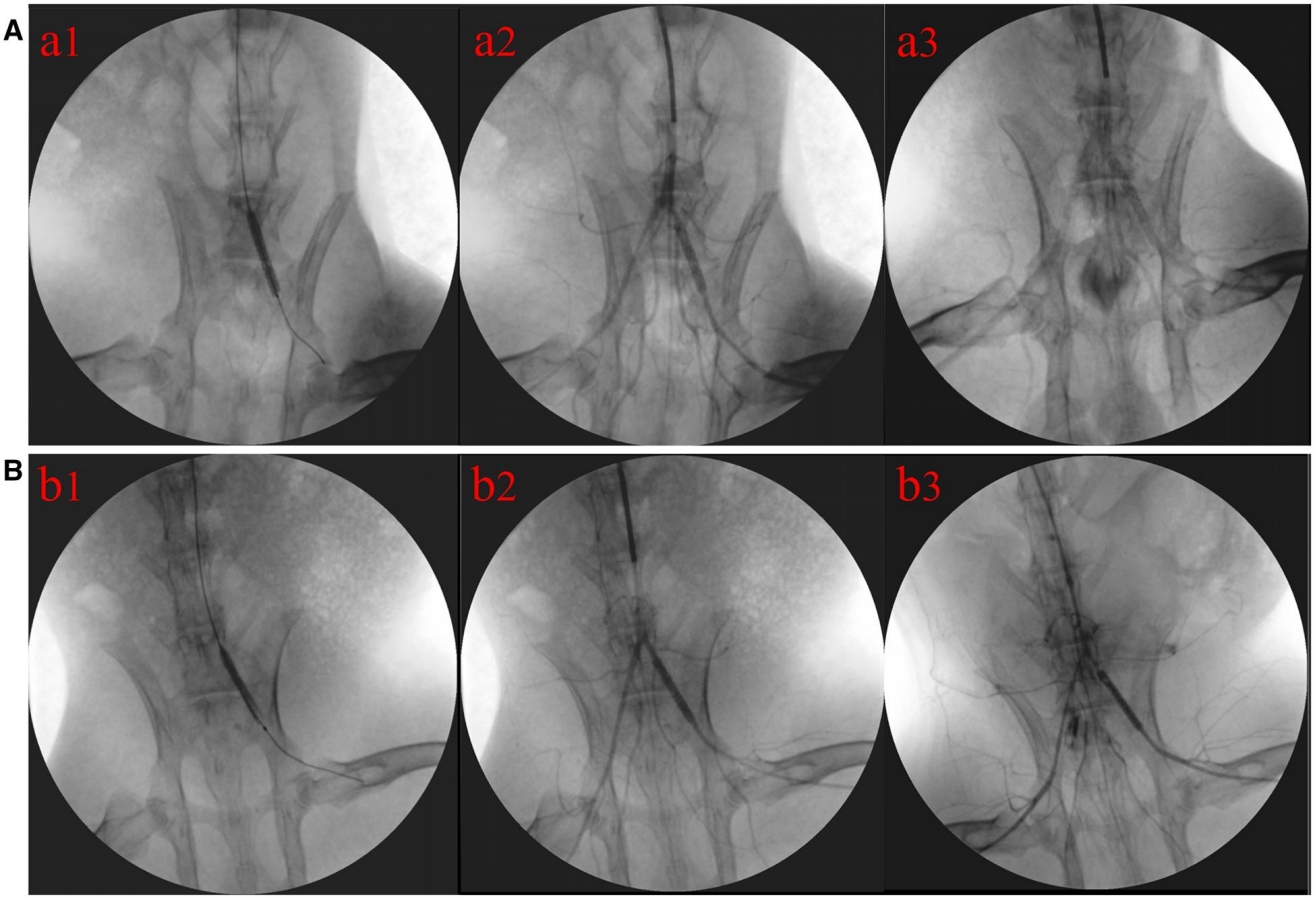
Stent implantation, immediate angiography after implantation and follow-up angiography 6 months after stent implantation of 316L (a1–a3) and 316L-Cu (b1–b3) stents, respectively.

A 5F Cobra catheter was placed at the end of the abdominal aorta following the described method to perform the bilateral iliac artery angiography. Subsequently, based on the angiography results, the balloon expandable stents were released onto the left iliac artery segment via injecting diluted contrast agent into the balloon (Figure 7, a1 and b1). Angiography was performed immediately after stent implantation, showing that the stents were accurately positioned and fully deployed, and the contrast agent could smoothly pass through the stents (Figure 7, a2 and b2). Six months after stent implantation, follow-up angiography was performed. The results demonstrated that both the position and morphology of 316L and 316L-Cu stents were normal, and there was no obvious stenosis inside the stents. The contrast agent could smoothly pass through both 316L and 316L-Cu stents (Figure 7, a3 and b3).

### Pathology analysis

**Figure 8.**
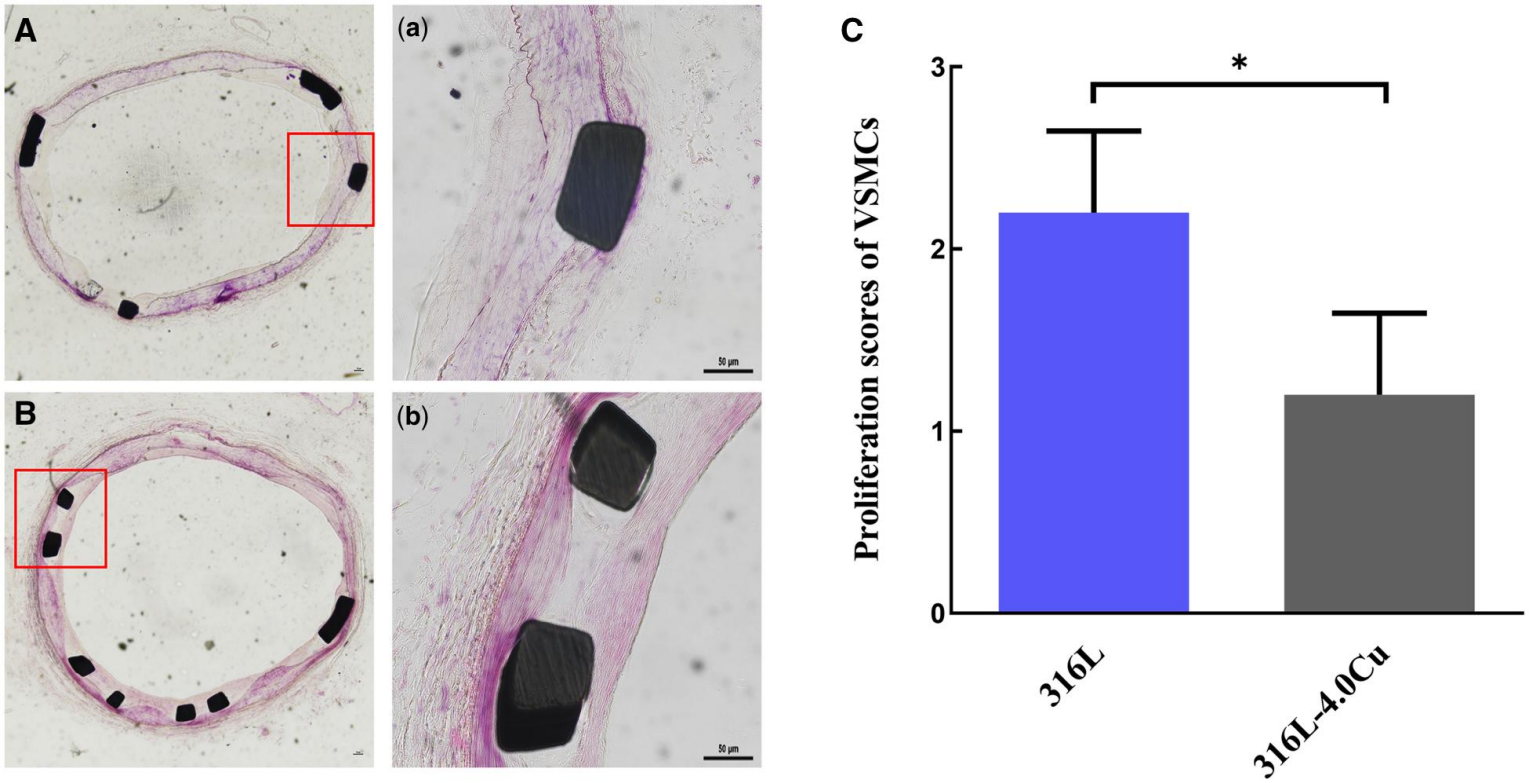
Photomicrographs of iliac artery sections 6 months after stent implantation. Hematoxylin–eosin stain of explanted 316L (**A**) and 316L-Cu (**B**) stents. (a) and (b) (×200) are the zoomed images indicated by the red squares in (A) and (B) (×40). (**C**) Quantitative morphometric analysis of proliferation scores of VSMCs.

The findings from hematoxylin–eosin (HE) staining demonstrated that 316L-Cu stent could effectively inhibit the proliferation of VSMCs. Compared to 316L stent (Figure 8A), there was no thickening and coverage of VSMCs observed at the site of 316L-Cu stent implantation (Figure 8B). The detailed structure is shown in Figure 8A and B, showing that the site of 316L stent implantation was completely covered by a layer of VSMCs. The proliferation scores of VSMCs on 316L and 316L-Cu stents were 2.20 ± 0.45, 1.20 ± 0.45, respectively. A statistically significant difference was observed in VSMC proliferation between 316L and 316L-Cu stents (Figure 8C).

## Discussions

ISR is one of the key issues that limit the efficacy of interventional therapy for lower extremity ASO. In order to solve this problem, enormous amounts of efforts have been made to develop new medical devices, including DESs and drug-eluting balloons (DEBs), even biodegradable stents. However, enthusiasm for these new devices has been dampened by concerns of long-term efficacy. The reason is that the drug coated on DES or DEB inhibits the proliferation of both VSMCs and endothelial cells, thus delaying the re-endothelialization and ultimately causing late thrombosis [29]. Numerous experimental and clinical studies have showed that under the premise of sufficient anticoagulation and antiplatelet therapy, the repair of endothelial cells (ECs) and the inhibition of the proliferation and migration of VSMCs are the core measures to reduce the incidence of ISR [30]. In addition, in the process of interventional therapy, balloons and guide wires often cause different degrees of damage to ECs, which makes the rare and fragile endothelial cells be repaired more slowly [31]. Copper (Cu), an indispensable trace element in the human body, could stimulate blood vessel formation [15, 32] and offer a strong antibacterial performance [33, 34]. The subsequent studies showed that Cu not only stimulated the proliferation and migration of ECs to promote blood vessel formation but also inhibited the proliferation and migration of VSMCs and thus prevented the formation of thrombosis [35, 36].

In this study, to develop a novel Cu-bearing stent material with the above biofunctions, different amounts of Cu (2.0, 2.5 and 3.5 wt.%) were added into a currently used stent material, cobalt-based alloy (L605), to fabricate the Cu-bearing cobalt-based alloy (L605-Cu) according to our previous studies. The biosafety and biological effects along with their related mechanism, such as the gene expressions of *bFGF*, *PDGF-B*, *HGF* and *Nrf2*-related downstream, genes as well as protein expressions of AKT and Nrf2, of HASMCs after co-culture with extracts of the 316L and L605 with and without Cu additions were investigated. Furthermore, to further investigate the influence of the Cu-bearing metals on the neointimal hyperplasia, an *in vivo* test on the 316L-4.0Cu stent manufactured by mature processes was conducted to provide direct scientific evidence for the biofunctionality of Cu-bearing metals. For the study of a novel biofunctional stent material, Cu is the key factor in the study. As the immersion time increased, the Cu ions released from 316L-4.0Cu and L605-3.5Cu were significantly higher than those from 316L, L605-2.0Cu and L605-2.5Cu, as shown in Figure 2. As the released Cu ions showed the bioactivity as expected, the biosafety, biological effects and the related mechanisms of the Cu ions on HASMCs were focused in this study.

Biosafety is the primary requirement of all medical devices, and it is necessary for the intravascular stent materials to possess good blood compatibility [37]. Previous studies have shown that the protein adsorption specificity and selectivity of materials have an important impact on the blood compatibility of materials [38]. It is generally believed that the albumin adheres to the surface of the materials can cause the formation of biological passivation film on the material surface, and inhibit the adhesion of platelets, thus preventing thrombosis [39, 40]. Adhesion of fibrinogen can activate and accelerate platelet adhesion and coagulation process [41]. In addition, Kaelble and Moacanin [42] pointed out that an increase in the polar component could promote plasma albumin adsorption on the material surface. The present study indicated that the surface hydrophilicity of the presently studied metals decreased with increase of Cu content in the metals, leading to both the bonding energy and the surface free energy of the metals to decrease.

Anti-clotting property is directly affected by the surface property of the implanted material. As the Cu addition was increased, the polar component of the surface tension was proportionally reduced, which should lead to more platelet adhesion, but there was no significant difference. Therefore, from the tendency of dynamic clotting time curve, the Cu-bearing metal with higher Cu content leaned to the blood clot. However, as the co-cultured time was prolonged, the released Cu ions had an inhibition effect on the clotting tendency. The more Cu added to the Cu-bearing metal, the more the Cu ions are released. Above all, both 316L-Cu stainless steel and L605-Cu alloy showed obvious anticoagulant performance. The dynamic clotting time test showed that 316L-Cu and L605-Cu all did not activate the endogenous clotting progress. Furthermore, no significant difference was found between 316L and 316L-Cu regarding hemolysis rate, as well as L605 and L605-Cu. Hemolysis rates of 316L, 316L-Cu, L605 and L605-Cu were all <5%, meeting the requirement on hemolysis for implant materials [43]. Previous studies have shown that the Cu-bearing metals could play an important role in promoting endothelialization and inhibiting VSMCs growth without relative side effect *in vivo* or *in vitro* [18, 20]. Moreover, in the present study, the Cu contents in 316L-Cu and L605-Cu were all in the range of 0–4.0 wt.%, which could play biological roles as biosafe as in the previous studies.

The migration ability of VSMCs plays a vital role in the development of ISR, which accelerates the proliferation of VSMCs to cover the stent [44]. Zhang *et al.* [45] demonstrated that growth medium containing either 250 or 500 μM of CuCl_2_ could significantly inhibit the migration of aortic smooth muscle cells *in vitro*. In the present study, the effect of Cu ions released from 316L-Cu and L605-Cu on transverse and longitudinal migrations of HASMCs was further examined. Transwell migration test demonstrated that 316L-4.0Cu and L605-3.5Cu could significantly inhibit the longitudinal migration of HASMCs, which should further reduce the incidence of ISR. Moreover, the scratch assay, which reflects the ability of transverse migration of cells, showed that 316L-4.0Cu, L605-2.5Cu and L605-3.5Cu could significantly inhibit the migration of HASMCs.

In addition, the proliferation of VSMCs is also crucial to the development of ISR [20, 45]. Unlike thrombosis on stents, ISR is caused by the proliferation of VSMCs and often cannot be solved by secondary interventional therapy, meaning that patients have to accept more traumatic surgical treatment [46]. In the present work, the results of CCK-8 assay and EDU assay indicated that both 316L-4.0Cu and L605-3.5Cu could inhibit the proliferation of HASMCs *in vitro*. Previously, Yang *et al.* [20]. showed that a suitable amount of Cu ions could continuously be released at the site of the stent implanted in vascular, and thus the proliferation of VSMCs could be reduced. In addition, the rapid development of surface coating technology can effectively avoid the complexity of processing copper-containing alloys, and can also have the biological functions of copper [47]. Fan *et al*. [17] developed nano Cu-MOFs with polydopamine-coated vascular stent and demonstrated that the proliferation of VSMCs and macrophage were inhibited by the nano Cu-MOFs-immobilized coating, reducing the neointimal hyperplasia *in vivo*. Furthermore, Wei *et al*. [48] fabricated an absorbable three-layer SA/HA vascular patch, which could effectively inhibit vascular neointimal hyperplasia.

The effect of Cu-bearing metals on the migration and proliferation of VSMCs has been studied; however, further studies on the related mechanism were rarely reported. Therefore, the mechanism of released Cu ions inhibiting the proliferation and migration of VSMCs was explored in this study. The migration and proliferation of VSMCs are a key event in restenosis, which is mediated by cytokines and growth factors released from the vascular wall [49]. Among them, both bFGF and PDGF-B contribute significantly to the migration of VSMCs in the development of ISR. The reason is that bFGF and PDGF-B are released by ECs, macrophages and VSMCs at the site of vascular injury, which often occurs during the stent placement [50]. Wang *et al.* [51] demonstrated that bFGF activated the phosphoinositide 3-kinase (PI3K)/protein kinase B (AKT) pathway and promoted the cell migration. Kim *et al.* [52] showed that PDGF-B promoted the cell migration and proliferation through activating AKT/extracellular-regulated kinase 1/2 (Erk1/2). In the present study, the Cu ions released from 316L-Cu and L605-Cu could down-regulate the levels of bFGF and PDGF-B, which led to the inhibition to migration of HASMCs. VSMCs proliferate significantly after balloon injury, and the expression of HGF increases during this process [53].

Moreover, Li *et al.* [54] demonstrated that cilostazol could promote the proliferation of VSMCs via up-regulating the expression of *HGF*. Stimulation with HGF leads to the activation of P13K, PKB/AKT, MEK and the MAP kinases Erk1 and -2, which mediates the migration of VSMCs [55]. Therefore, HGF is one of the important factors affecting the occurrence of ISR. In addition, a previous study found that HGF is a Cu-binding protein [56], which inhibits proliferation and migration of cells when combined with Cu [57]. Similarly, in this study, the Cu ions released from 316L-Cu and L605-Cu reduced *HGF* expression, resulting in the inhibition of proliferation and migration of HASMCs.

In addition, Cu-containing materials could activate eNOS to secrete NO [58]. The NO synthesized by eNOS plays a key role in inhibiting the proliferation and migration of HASMCs [59, 60]. However, our previous study found that Cu-bearing metals could not stimulate the NO secretion or mRNA expression of *eNOS*, which may be due to the following reasons. On the one hand, the amount of Cu ions released is insufficient for enhancing NO secretion or mRNA expression of *eNOS*, and on the other hand, Cu-containing materials may take longer to play their role [21]. In this study, the inhibition effect of 316L-4.0Cu and L605-3.5Cu on the migration and proliferation of HASMCs arose from inhibiting the AKT/Nrf2/ARE signaling pathway, which is difficult to distinguish the similar biological function produced by eNOS/NO. Moreover, Li *et al*. [61] found that the copper-bearing stainless steel could promote the apoptosis of coronary artery smooth muscle cells through increasing the expression of Fas protein. Inflammation has been identified as a key factor contributing to VSMC proliferation and thrombosis. Li *et al*. [19] showed that 316L-Cu stainless steel could suppress the inflammation caused by endothelial dysfunction via blockading the inflammatory factors (TNF-α, IL-1β, 6, 8), which may reduce the proliferation of VSMCs. Therefore, Cu-containing materials may play a biological role in inhibiting the proliferation and migration of HASMCs through a variety of mechanisms.

Previous studies showed that the AKT signaling pathway was closely related to various cellular activities, including proliferation, migration and differentiation [62]. Recent studies indicated that the AKT signaling pathway played a key role in the development of atherosclerosis obliterans [63]. Linton *et al.* [64] found that inhibition of AKT signaling significantly decreased the viability and proliferation of monocytes and macrophages with suppression of atherosclerosis. Zhang *et al.* [65] indicated that repression of the PI3K/AKT/mTOR pathway could alleviate the proliferation, migration and phenotypic switch of VSMCs. Furthermore, Wu *et al.* [66] demonstrated that zinc-doped copper oxide nanocomposites disturbed cell growth by inhibiting AKT and Erk1/2 activation. In this study, the Cu ions released from 316L-Cu and L605-Cu might reduce the proliferation and migration of HASMCs via inhibiting the expression of AKT.

Nuclear factor erythroid-2 related factor 2 (Nrf2) is a member of the Cap’n’Collar (CNC)-bZIP transcription factor family, which plays a key role in the oxidative stress response [28]. Many studies showed that Nrf2 exhibited pro- and anti-atherogenic effects in animal models [67, 68]. An early study has reported that global Nrf2 knockout inhibited the intimal hyperplasia of wire-injured femoral arteries in mice [69]. Recently, Li *et al.* [23] demonstrated that Nrf2 deficiency reduced atherosclerotic plaque burden and inhibited the proliferation and migration of VSMCs *in vitro* and *in vivo*. In this study, the expression of Nrf2 was decreased by the Cu ions released from 316L-Cu and L605-Cu, and the level of Nrf2 was continuously decreased by L605-Cu as the Cu content increased, which indicated that 316L-Cu and L605-Cu might reduce the proliferation and migration of HASMCs via inhibiting the expression of Nrf2.

*HO-1* is a downstream gene that is activated by *Nrf2* [70]. Earlier studies have elucidated that Nrf2 also has significant effects on VSMCs via ARE, NQO1 and other signaling pathways [71]. In addition, some studies suggested the opposing effects of Nrf2 signaling in the regulation of GCLC and GCLM expressions in old and diabetic animals [72, 73]. It was also found in this study that the Cu ions released from 316L-Cu and L605-Cu inhibited the protein expression of Nrf2 and lowered its downstream gene expression. Wang *et al.* [27] found that exposure of ARPE cells to PI3K inhibitors caused a decrease in Nrf2 activity. Furthermore, to investigate the possibility that the AKT/Nrf2/ARE signaling pathway might be involved in the inhibition of migration and proliferation of VSMCs by the Cu-bearing metals, it was found that 316L-Cu significantly decreased the expressions of phospho-AKT, Nrf2 and GCLM in HASMCs compared with 316L. L605-Cu significantly decreased the expressions of phospho-AKT, Nrf2 and HO-1, NQO1, GCLC and GCLM in HASMCs compared with L605. And SC79 (an activator of AKT) and TBHQ (an inducer of Nrf2) were used to activate the AKT/Nrf2 signaling pathway. We found that SC79 treatment could significantly increase the expressions of phospho-AKT and GCLM in 316L group, and also could significantly increase the expressions of GCLC and NQO1 in 316L-Cu group. SC79 treatment could significantly increase the expressions of phospho-AKT, GCLC, GCLM, NQO1 and HO-1 in L605 group, and also significantly increase the expressions of phospho-AKT, GCLC, NQO1 and HO-1 in L605-Cu groups. Moreover, TBHQ treatment could significantly increase the expressions of Nrf2, GCLM and NQO1, except phospho-AKT in 316L group. TBHQ treatment could greatly increase the expressions of Nrf2 and NQO1 proteins in 316L-Cu. After treatment with TBHQ, GCLC, GCLM and HO-1 expressions tended to increase in 316L-Cu group. TBHQ treatment could significantly increase the expressions of Nrf2, GCLC, GCLM, NQO1 and HO-1, except phospho-AKT in L605 group, and TBHQ treatment could significantly increase the expressions of Nrf2, GCLC, NQO1 and HO-1, except phospho-AKT in L605-Cu group. All these results indicated that 316L-Cu and L605-Cu inhibited the proliferation and migration of HASMCs via the AKT/Nrf2/ARE pathway, and L605-Cu showed better biological reaction.

Based on the above promising results, an *in vivo* study was conducted to further evaluate the ability of Cu-bearing metals to inhibit neointimal hyperplasia. In the present study, 316L and 316L-Cu stents were used to comparatively study the effect of Cu-bearing metal stents *in vivo.* An angiography was performed 6 months after the stents were implanted, showing that there was no obvious stenosis inside the 316L and 316L-Cu stents. However, proliferation scores of VSMCs based on the pathology analysis demonstrated that the 316L-Cu stent could effectively inhibit the formation of VSMC layer *in vivo*, although this study was not conducted on a restenosis model. Thus, it will be necessary in the future to establish an animal model of ISR for a more systematic study, and the animal study of the L605 stent is also needed in the future.

## Conclusion

The effects of Cu-bearing metals, including 316L-Cu stainless steel and L605-Cu cobalt-based alloy, on migration and proliferation of VSMCs were studied through *in vitro* and *in vivo* experiments. It was found that the Cu-bearing metals obviously inhibited both the proliferation and migration of VSMCs with no cytotoxicity and good blood compatibility. The mRNA expressions of *bFGF*, *PDGF* and *HGF* were inhibited by Cu-bearing metals. The mRNA expressions of *Nrf2* and *Nrf2*-related downstream genes, such as *GCLC*, *GCLM*, *NQO1* and *HO1*, were also suppressed by Cu-bearing metals. The inhibition effects of Cu-bearing metals on the migration and proliferation of VSMCs arose from inhibiting the AKT/Nrf2/ARE signaling pathway. Furthermore, the animal test further proved that the 316L-Cu stent could effectively inhibit the proliferation of VSMCs.
